# The cost-utility of school-based first permanent molar sealants programs: a Markov model

**DOI:** 10.1186/s12903-019-0990-3

**Published:** 2019-12-30

**Authors:** Gerardo Espinoza-Espinoza, Gilda Corsini, Rubén Rojas, Rodrigo Mariño, Carlos Zaror

**Affiliations:** 1 Department of Public Health, Faculty of Medicine, Universidad de La Frontera, Temuco, Chile; 2 Center for Research in Epidemiology, Economics and Oral Public Health (CIEESPO), Faculty of Dentistry, Universidad de La Frontera, Temuco, Chile; 3 Center for Research and Innovation in Clinical Dentistry (CIDIC), Faculty of Dentistry, Universidad de La Frontera, Temuco, Chile; 4 School of Health and Related Research (ScHARR), The University of Sheffield, Sheffield, South Yorkshire UK; 5 Melbourne Dental School, The University of Melbourne, Parkville, Victoria Australia; 6 Department of Pediatric Dentistry and Orthodontics, Faculty of Dentistry, Universidad de La Frontera, Manuel Montt #112, Temuco, Chile

**Keywords:** Pit and fissure sealants, Dental caries, Prevention, Cost-effectiveness analysis

## Abstract

**Background:**

Evidence of the cost-effectiveness of school-based first permanent molar sealants programs is not yet fully conclusive. The aim of this study was to determine the incremental cost-utility ratio (ICUR) of school-based prevention programs for the application of sealants in molars of schoolchildren compared with non-intervention.

**Methods:**

A cost-utility analysis based on a Markov model was carried out using probability distribution. The utility was measured in quality-adjusted tooth years (QATY). The assessment was carried out from the public payer’s perspective with a six-year time horizon. Costs and benefits were discounted at 3% per year. Only direct costs were evaluated, expressed in Chilean pesos (CLP) at 7th May at 2019 values (exchange rate USD = CLP 681.09). Univariate deterministic sensitivity analysis and probabilistic analysis were carried out.

**Results:**

After a six-year follow up, the cost of sealing all first permanent molars was found to be higher than non-intervention, with a mean cost difference of USD 1.28 (CLP 875) per molar treated. The “seal all” strategy was more effective than non-intervention, generating 0.2 quality-adjusted tooth years more than non-intervention. The ICUR of the “seal all” strategy compared to non-intervention was USD 6.48 (CLP 4,412) per quality-adjusted tooth years. The sensitivity analysis showed that the increase in caries was the variable which most influenced the ICUR.

**Conclusions:**

A school-based sealant program is a cost-effective measure in populations with a high prevalence of caries.

## Background

The teeth most susceptible to dental caries are the first permanent molars (FPM), due to their occlusal anatomy which favors the retention of bacterial plaque, their position in the arch, which prevents adequate oral hygiene, and the immaturity of the teeth at the time of eruption [1]. The prevalence of caries in FPM can be as high as 90% in populations with poor socio-demographic characteristics, with the occlusal surface most affected [2, 3]**.**

One way of preventing pit and fissure dental caries in first permanent molars, is by applying resin-based sealants [2, 3]. These have proven to be highly effective, resulting in a decrease of caries incidence, from 86% in the first year, to 59% 4 years following application [4, 5]. However, various factors may influence the success of sealants in a school-based prevention program, such as the prevalence of caries in the population, the age of the patients, the subject’s risk of caries, and fissure sealant retention [6, 7].

Knowledge of the effectiveness and safety of any given intervention by itself, is not enough to decide on its implementation. Cost-effectiveness, as well as the political, organizational, social, ethical and legal impacts must be considered, especially when applied within the public health context [8]. Therefore, economic evaluations are an important factor for the implementation of any preventive program. The evidence provided by economic evaluation regarding sealant treatment at a school-based prevention program, is as of yet inconclusive [9]. However, greater cost-effectiveness has been shown in school populations with a high risk of caries [10, 11].

Most studies carried out thus far have used average values ​​for transition probabilities, generating a base case in which hypothetically all children have the same experience. These studies did not however, consider the variability between children [10, 12, 13]. Consequently, and in order to improve this approach, we decided to develop a model based on probability distribution, in which the transition probabilities vary between individuals**.** This model is a simulation that represents the full spectrum of possible values, of a given transition probability. These transition probabilities are reflected in frequency densities; when this variability is considered, the model approximates the actual event more precisely [14].

Furthermore, it is important to include patient preferences in the economic evaluations, in relation to treatment efficacy and the alternatives. This translates into reduced distance in the assessment between the sound tooth and the filled tooth, which renders the analysis more similar to the real setting [15].

The aim of this study was to determine the incremental cost-utility ratio (ICUR) of a school-based prevention program, applying dental sealants in first permanent molars in schoolchildren, compared with non-intervention from the payer’s perspective and after 6 years for the conditions prevailing in Chile. Further evidence on the cost utility of dental sealants would be useful to practitioners, as well as public health planners who need to decide about dental public health programs and future initiatives. Furthermore, according to Griffin and her collaborators, the overall economic benefit of dental sealants would not differ across jurisdictions [16]. In this way, this study will provide economic information for subsequent studies on the benefits of dental sealants in children and would assist in the implementation of health policies and preventive and treatment programs in both developed and developing countries.

## Methods

A cost-utility study was carried out based on a Markov model to represent the different states of health derived from the implementation of a school-based dental sealant program in children, where the intervention and control cohorts were modeled to represent the reality.

The assessment in this study was carried out from the public payer’s perspective (i.e., local municipality). Therefore, only direct costs, such as human resources and supplies were included.

This study was prepared according to Consolidated Health Economic Evaluation Reporting Standards (CHEERS) [17]. In this model, probability distribution was used to take into account the variability of the individuals in the analysis.

**Fig. 1:**
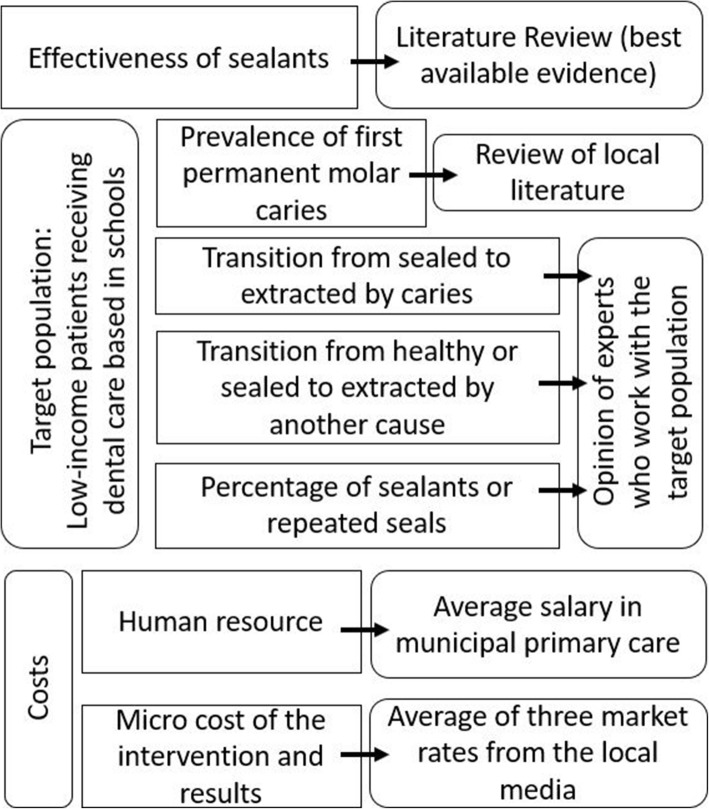
Diagram with the inputs used in the model

Figure 1 shows a diagram of the inputs used in the model and the different methods used to obtain the respective values.

### Population and setting

In Chile, 278 districts throughout the country have dental units in the public schools, funded by the Chilean government. This program provides comprehensive, systematic and scheduled dental care for the students, from kindergarten (approximately 4 years of age) until graduation from basic and middle school education (approximately 14 years of age). Standard procedures include preventive measures such as fluoride varnish and sealants application, recommendations to use fluoridated toothpaste, oral hygiene instructions and dietary counseling. Treatments include restoration with resin and dental extraction. From 6-years-old and after the complete eruption of permanent molars, the program considers sealing the FPMs of all children. Therefore, the hypothetical population included in this model considered 6-year-old children of low socioeconomic status, and with high risk of caries in first permanent molars from public schools with a dental unit, with erupted, sound first permanent molars. The intervention was modeled considering that it would be carried out in public schools in urban areas with access to drinking water containing optimum fluoridation levels, and to school-based sealant programs.

### Interventions

The “seal all” intervention included in the theoretical model consisted of sealant application in all sound FPMs, regardless of caries risk. All the assumptions incorporated into the model, both for effectiveness and costs, considered that the modeled strategy had the following characteristics: The resin-based sealants were applied on first molars erupted with cotton roll isolation, by dentists of the public health system, with 1 to 10 years of clinical experience and with the help of a dental assistant. The dental unit was fully equipped including suction unit. Interproximal caries lesions were not assessed. The modeled strategy included children receiving follow-up check-ups every 24 months, at the ages of 8, 10 and 12 years. During each follow-up check-up, the sealant was repaired for partial or total loss as deemed necessary.

The comparison strategy was “no seal” meaning non-intervention with sealants. In both strategies when the patient presented caries, the model considered that the lesion was filled with resin composite; when a tooth extraction was warranted, it was carried out. Restorations and extractions were performed by a dentist and a dental assistant at a public dental clinic.

### Time horizon

The time horizon was 6 years. The first year was the baseline, when children were 6 years old, and the last was that of the final examination at 12 years, with two intermediate check-ups.

### Discount rate

A discount rate of 3% per year was used for costs and effects, as recommended in the Methodological Guide for the Economic Assessment of Health Interventions in Chile [18].

### Effectiveness measure

The effectiveness measure of the intervention in this study was the prevalence of caries in FPM, incorporated into the model as the probability of caries at the ages of 6, 8, 10 and 12 years.

Sealant effectiveness results were obtained from a Cochrane review, which included a metanalysis of 3.620 patients. The review determined effectiveness of 78% (CI 95%: 66–85%) after 24 months, and 60% (CI 95%: 49–69%) after 48 months [6]. To determine effectiveness of the resin-based sealants after 72 months, a variation of 6% per year in adolescents was considered, as reported in the literature (48%; CI 95%: 37–57%) [19].

To determine the prevalence of caries in the comparator, a systematic search in Medline, Lilacs, SciELO and Cochrane databases was carried out. Studies were included if they assessed the prevalence of caries in FPM in children aged 6–12 years, in Chile and Latin American countries with similar socio-cultural characteristics. Three studies reported the prevalence of caries in FPM at the age of 6 years, [20–22] another three at the age of 12 [23–25], and one reported prevalence at the age of 8 and 10 years [26]. When necessary, weighted means were calculated according to the number of children examined. Therefore, the prevalence studied in the untreated population was estimated at 25% in children aged 6 years, while prevalence for the other ages was determined under the assumption that the increase in caries (percentage increase of prevalence in each Markov cycle) was continuous over time [9, 27].

A plot points graph was designed using all of the available data. In the “x-axis” ages were recorded, and prevalence was observed at different ages in the “y-axis”. A straight line was achieved intersecting both, representing the increase of caries in the population. The increase in the prevalence of caries in the untreated population was 10.3% during each two-year cycle. The prevalence values used in the models were 25% at 6 years; 35.3% at 8 years; 45.6% at 10 years; and 55.9% at 12 years (See Table 1).

**Table 1:** Parameters, probabilities and distributions used in the model

Items	Seal everyone or Seal All (%)	No Seal (%)	Type of distribution used
Effectiveness of sealants (CI 95%)^a^	2 Y: 78 (66–85)	–	A probability table was used
	4 Y: 60 (49–69)	–	
	6 Y: 48 (37–57)	–	
Prevalence of caries in first permanent molars (range for sensitivity analysis)^b^	6 YO: 0	6 YO: 25	Normal
	8 YO: 8 (± 2)	8 YO: 35.3 (± 2)	Normal
	10 YO: 18 (± 2)	10 YO: 45.6 (± 2)	Normal
	12 YO: 29 (± 2)	12 YO: 55.9 (± 2)	Normal
First permanent molars lost in each 2-year cycle ^c^			
Cavities	1 in one thousand	1 in one thousand	Beta
Other causes	1 in ten thousand	1 in ten thousand	Beta
Reseal rate per cycle (Range for sensitivity analysis)^c^	3 (0–13)	–	Beta
Re-filling rate per cycle (Range for sensitivity analysis)^d^	1 (0–14)	1 (0–14)	Beta

*Y* Years since application, *YO* Years old

^a^Cochrane systematic review [6]

^b^Local Evidence from Chile and Latin America [21, 22, 24–26]

^c^Survey of 10 experts working in school-based dental clinics

^d^Survey of 10 experts working in school-based dental clinics and literature review for the variability [9, 28, 29]

Since the prevalence of caries in FPM for the intervention population was not found in the published literature, it was estimated indirectly considering two aspects. Firstly, the effectiveness of caries prevention, and secondly, the prevalence of caries in FPM in the non-intervention population. Subsequently, the percentage of caries averted was subtracted from the prevalence in the non-intervened population, depending on the effectiveness of intervention for each age group.

### Estimating the utility

Utility was calculated using quality-adjusted tooth years (QATY) [30–33]. QATY is a measure of dental health analogous to quality-adjusted life-year, that provides an outcome measure which could be compared across treatments and across clinical problems. The assumption underlying QATY is that teeth with any health status (painful, poor aesthetic, filled, missing, etc.), are not equivalent to sound teeth without that health conditions (e.g., sound), thus it provides an adjustment to account for this difference [33]. A value of 1 QATY was assigned to teeth which were sound or sealed without evidence of caries after 6 years; 0.81 QATY for teeth that required fillings; and 0 QATY for extracted teeth [34].

### Resources and costs

Only direct costs were assessed in the present study. The cost of interventions and treatment of new caries lesions were determined by micro-costing, including the necessary equipment, instruments and supplies for each intervention, and the cost of human resources required for each procedure. Estimates for the cost of equipment, instruments and supplies were obtained from quotes of three different commercial dental suppliers, and a mean value per patient was obtained for each item. A ten-year obsolescence limit was considered for the equipment, that is the average time determined by municipalities to renew their equipment.

The cost of human resources was obtained by multiplying the cost of one minute of the salary of the dentist and his assistant, multiplied by the average number of minutes it takes to perform the intervention. The salary was calculated to be the mean, between the salary paid to the employees with the least seniority in the workplace (one year) and senior employees with the longest period of employment (10 years). The costs for health care worker wages were obtained from a municipality standard salary scale, representative of the Chilean population [35]. To calculate the cost per minute, the average salary was divided by the number of minutes worked. The time of each intervention was determined according to the National Board for Scholarships and School Assistance (JUNAEB) which standardizes guidelines for the dental units in schools [36], which corresponds to 15 min for sealants, and 30 min for seals (See Table 2). The building costs were not considered. For further details, see Additional file 1.

**Table 2:** Direct costs used in the model

Intervention	Item	Time required	Value per minute	Cost per molar intervened (cost range
Sealants	Dentist	15 min	USD 0.236	USD 3.546 (3.039–4.142)
	Assistant	15 min	USD 0.066	USD 0.991 (0.846–1.269)
	Supplies	see Additional file 1		USD 0.473 (0.433–0.532)
	Clinical site preparation	5 min	USD 0.066	USD 0.33 (0.305–0.374)
	Equipment and instruments	15 min	USD 0.004	USD 0.066 (0.059–0.079)
Total				USD 5.406 (4.682–6.394)
Filling (composite	Dentist	30 min	USD 0.236	USD 7.092 (6.089–8.274)
	Assistant	30 min	USD 0.066	USD 1.982 (1.691–2.539)
	Supplies	see Additional file 1		USD 1.593 (1.436–1.752)
	Clinical site preparation	5 min	USD 0.066	USD 0.33 (0.305–0.374)
	Equipment and instruments	30 min	USD 0.004	USD 0.132 (0.117–0.147)
Total				USD 11.129 (9.639–13.083)
Extraction	Dentist	10 min	USD 0.236	USD 2.364 (2.405–2.754)
	Assistant	10 min	USD 0.066	USD 0.661 (0.561–0.846)
	Supplies	see Additional file 1		USD 0.59 (0.532–0.639)
	Clinical site preparation	5 min	USD 0.066	USD 0.33 (0.305–0.374)
	Equipment and instruments	10 min	USD 0.004	USD 0.044 (0.04–0.048)
Total				USD 3.989 (3.462–4.663)
Oral exam	Dentist	15 min	USD 0.236	USD 3.546 (3.039–4.142)
	Assistant	15 min	USD 0.066	USD 0.991 (0.846–1.269)
	Supplies	see Additional file 1		USD 0.216 (0.197–0.226)
	Equipment and instruments	15 min	USD 0.004	USD 0.066 (0.059–0.079)
Total oral exam				USD 4.603 (4.142–5.72)
Total Molar exam	Divided by 24 teeth			USD 0.192 (0.173–0.236)

### Currency, date and conversion costs

The study was conducted in Chilean Pesos to May 7, 2019 and adjusted with the consumer price index as needed. The values were reported in US dollars and the conversion was performed using the exchange rate of the Central Bank of Chile (1 USD = CLP 681.09).

### Model

The Markov model was chosen because it allows modelling of the natural course of events that represent the various possible health conditions. The Markov model assumed that patients resided in one of a finite number of health states at any point in time and made transitions between those health states over a series of discrete time intervals or cycles [37]. Therefore, it considered the probability of a FPM with caries lesions.

A probabilistic model was chosen since it allows a better simulation of the current reality with the spectrum of values that can assume the variables in each of the cases. It also allows observation of how that variability can affect the cost-utility relationship and carry out an analysis of multivariate sensitivity.

**Fig. 2:**
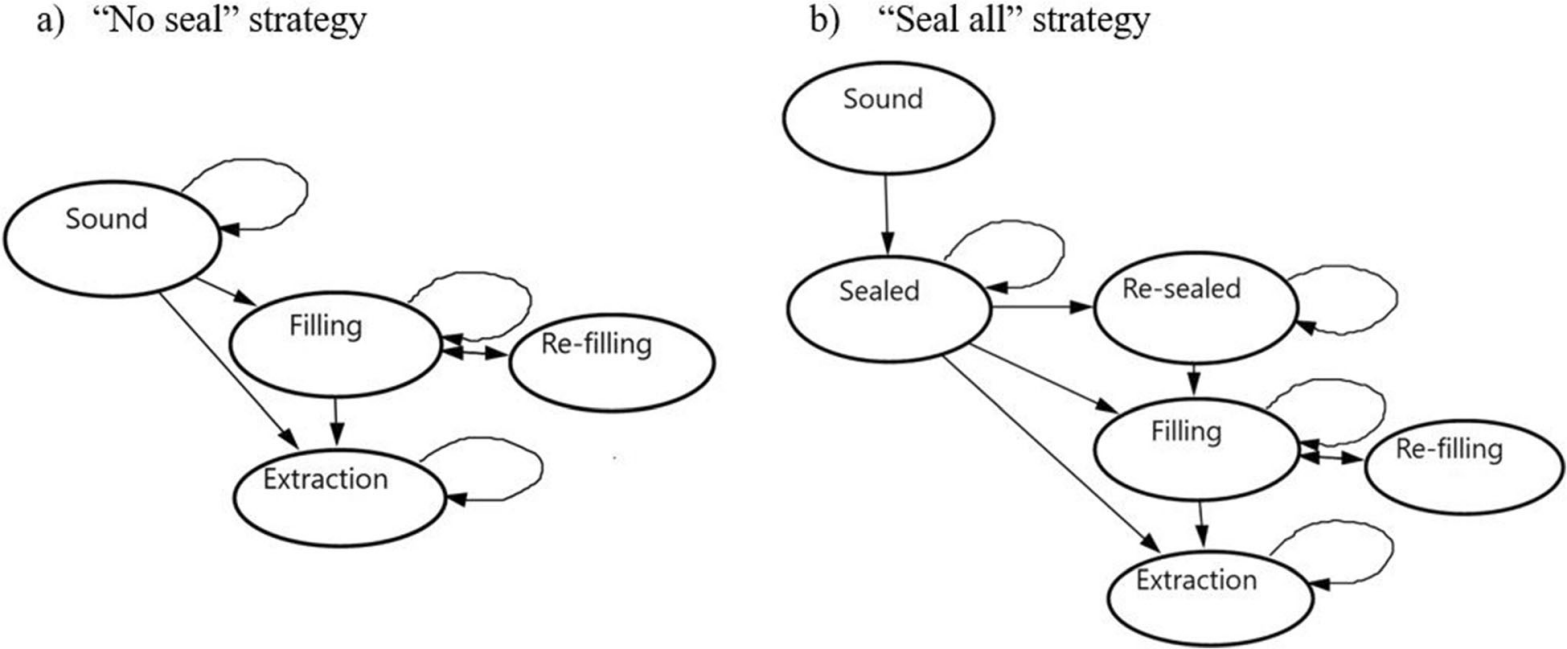
Markov models for each strategy **a**) Transition diagram of not treat **b**) Transition diagram of the option with treatment

The model was validated by a pediatric dentist and by an economist, both with extensive training in health technology assessment. Figure 2 shows a diagram of the model for each of the strategies.

### Assumptions

In this study, four assumptions were made, which were not based on the literature, but on the experience of ten dentists working in school-based dental services in each two-year cycle; a) dental sealant failure rate (replaced) would be 3%; b) 1% of fillings in teeth would be replaced; c) extractions due to caries would be performed at a rate of 1:1000; and d) extractions due to other causes that take place at a rate of one per ten thousand (See Table 1).

### Cost-utility analysis

To assess the ratio of costs to utility the quality-adjusted tooth year (QATY) was used [31]. In this analysis, the incremental cost-utility ratio (ICUR) was calculated, which corresponds to the division of incremental costs and incremental effects, to obtain the cost per QATY.

In the absence of a threshold of QATY willingness to pay, a reasonable threshold of USD 29.36 (CLP 20,000) was considered. This represents the cost for a tooth filling in the Chilean public oral health care system in March 2018 [38].

### Sensitivity analysis

To examine changes in the cost-utility ratio results under the effects of potential changes in the base values of the model parameters, a two-stage sensitivity analysis was carried out:

- Deterministic sensitivity analysis: In this one-way analysis, the average value of each variable is taken as a base case, and different parameters are used for the sensitivity ranges depending on the variable. For effectiveness, confidence intervals were considered [6]; for caries incidence a variation of ±2%, was explored; for reseal rate a range of 13% was considered to take into account the clinicians’ variability [9]. In reference to the re-filling rate, a range of between 0 and 14% was applied [9, 28, 29]. For the remainder of the parameters a variation of ±20% of the base value was considered. Finally, the results were classified from highest to lowest and represented in a tornado diagram.
- Probabilistic sensitivity analysis: Explores the simultaneous variation in the values of the variables analyzed, probability distributions (Table 1) were assigned to each of the parameters. Through a Monte Carlo simulation process, 1.000 iterations were performed, considering a random value within each distribution, and generating a result of incremental cost-utility ratio (ICUR). This allowed us to estimate how the simultaneous variation of the parameters would affect the utility cost estimator.

The data were analyzed using TreeAge pro 2019 software.

## Results

The estimated effectiveness for the prevalence of caries in FPM for the intervention population was 8% 2 years after sealant application, 18% 4 years after application and 29% 6 years after the intervention (Table 1).

Table 2 shows the detail of the costs considered for each intervention and its consequences. The mean cost of sealants was USD 5.4 (CLP 3682), ranging from USD 4.68 to USD.6.39 The mean value used for resin restoration was USD 11.13 (CLP 7580), ranging from USD 9.63 to USD.13.08. The mean value used for extractions was USD 3.98 (CLP 2717), ranging from USD 3,46 to USD.4,66 The mean value used for molar examination was USD 0.19 (CLP 131), ranging from USD 0.173 to USD 0.236 (See Additional file 1).

### Incremental costs and outcomes

**Fig. 3:**
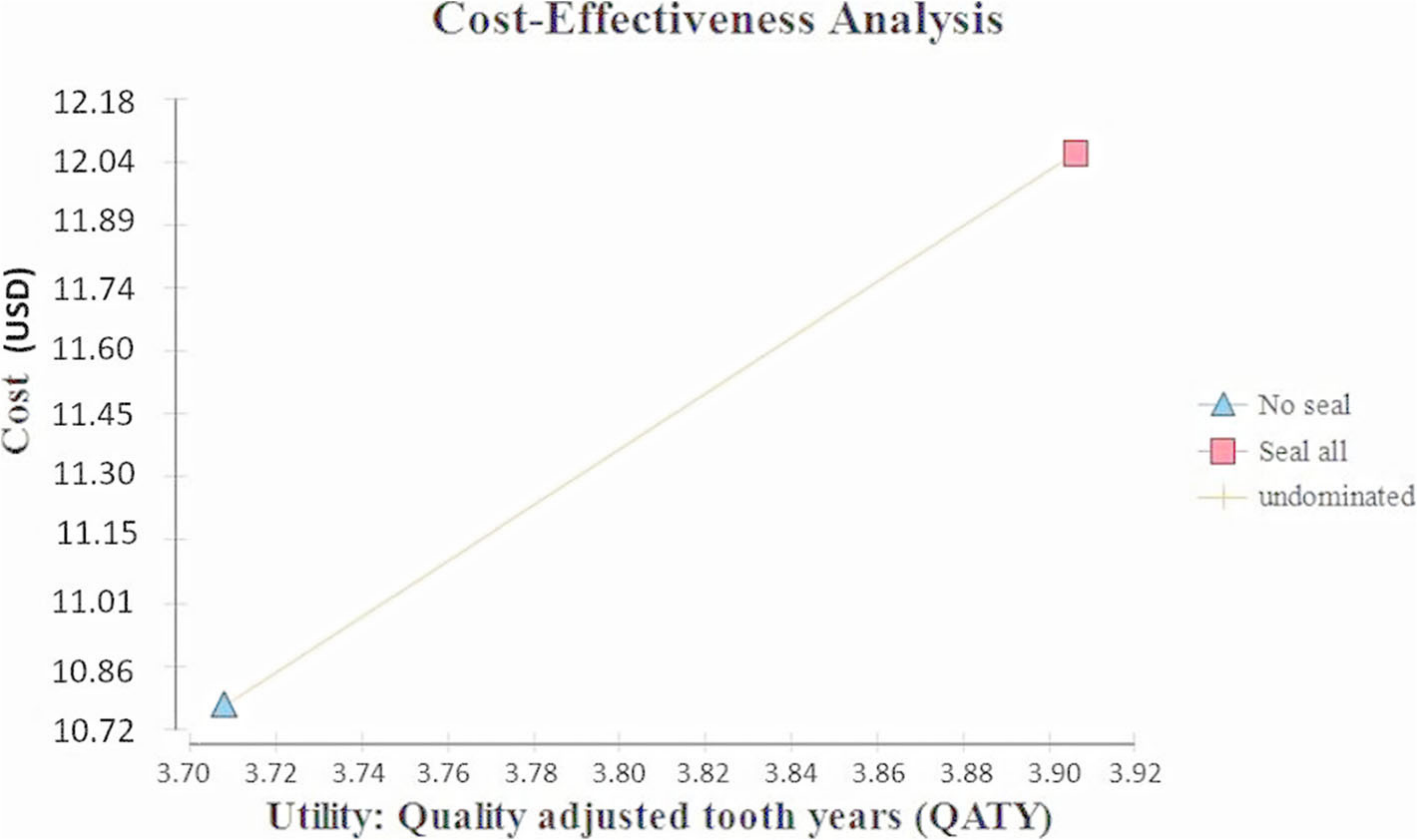
Cost-utility plane

If the “seal all” strategy is compared with non-intervention (“no seal”), the cost difference for each molar with intervention is USD 1.28 (CLP 875), over the six-year follow-up. The “seal all” strategy is more favorable, with a positive difference of 0.2 QATY. The ICUR of the “seal all” strategy as compared to non-intervention was USD 6.48 (CLP 4412) per QATY (Table 3). Figure 3 shows the cost-utility ratio of the “seal all” strategy vs. “no seal”, using the cost-utility plan.

**Table 3:** Incremental cost-utility ratio applying a Monte Carlo simulation

Strategies	Costs (USD)	Incremental cost (USD)	Utility (QATY)	Incremental utility	Incremental utility cost ratio
No seal	10.77		3.71		
Seal All	12.06	1.28	3.91	0.2	6.48

### Characterizing uncertainty

**Fig. 4:**
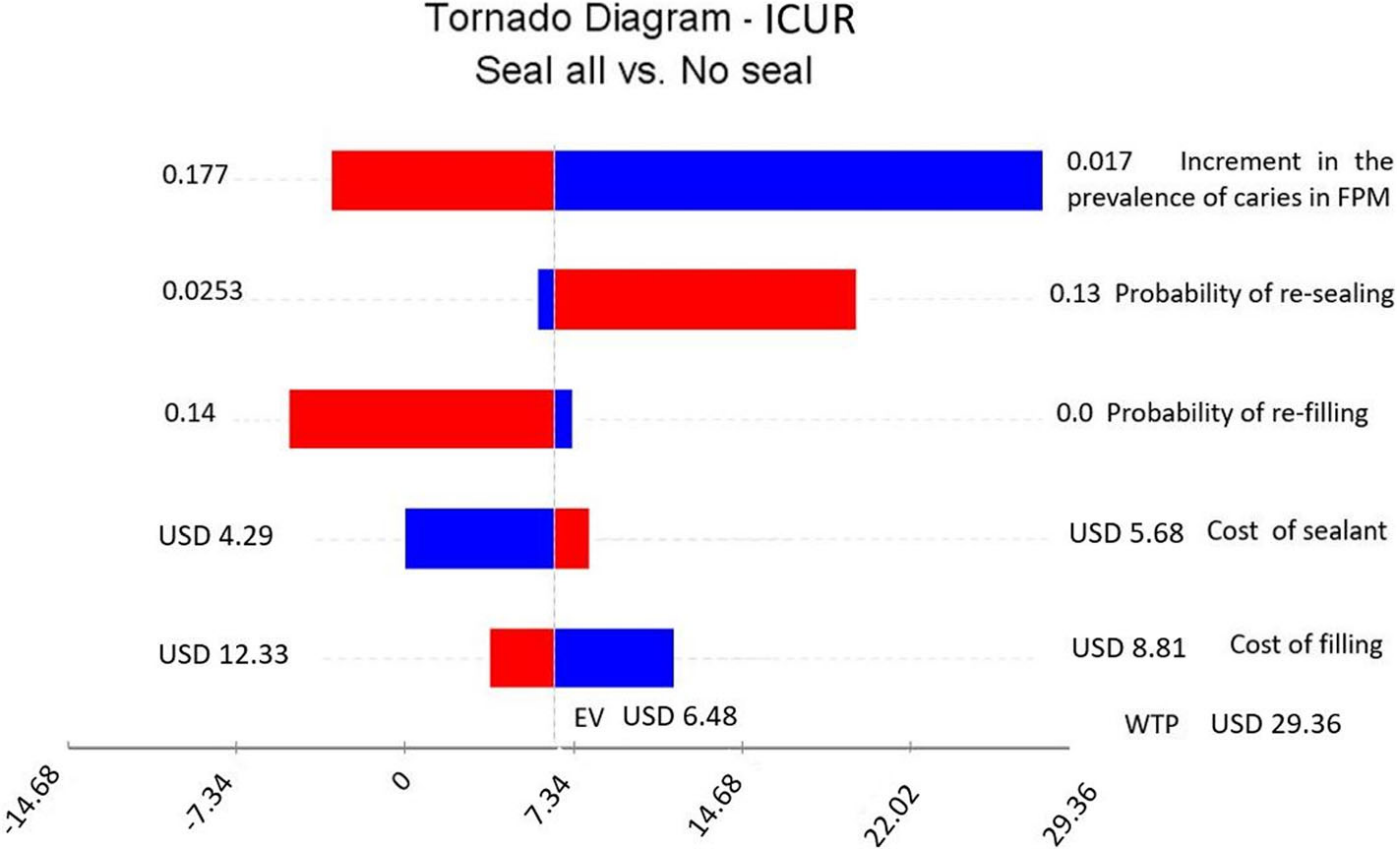
Tornado diagram for univariate sensitivity analysis

The deterministic sensitivity analysis is summarized in the tornado diagram (Fig. 4). The variable that most influences ICUR, is the increment in the prevalence of caries in FPM in the non-intervened population. The base case was calculated by estimating this increment in the prevalence of caries in FPM at 10.3% in each two-year cycle. In populations where this increment of caries prevalence is greater than 17%, the “seal all” strategy was dominant, while in populations where the caries increment is less than 5%, the ICUR doubles. It should be noted that if the increment is 2% or less in each two-year cycle, the ICUR is four times greater.

Other variables that cause uncertainty in the ICUR were the probability of re-sealing and re-filling. In the first variable the operator-effect influenced the success of sealant retention: between 0.025 and 13% of the sealants applied in the previous two-year cycle, required re-sealing. This resulted in a variation in the ICUR ranging from USD 5.85 (CLP 3988) to USD 19.71 (CLP 13,430). In relation with the re-filling, the “seal all” strategy became dominant (more effective and cheaper) when re-filling was 14%. On the other hand, when this variable is 0 the ICUR value increase slightly (USD 7.28; CLP 4959).

The other variables had little influence on the variation of the ICUR. The “no seal” strategy was not dominant in any scenario, reflecting the robustness of the results.

**Fig. 5:**
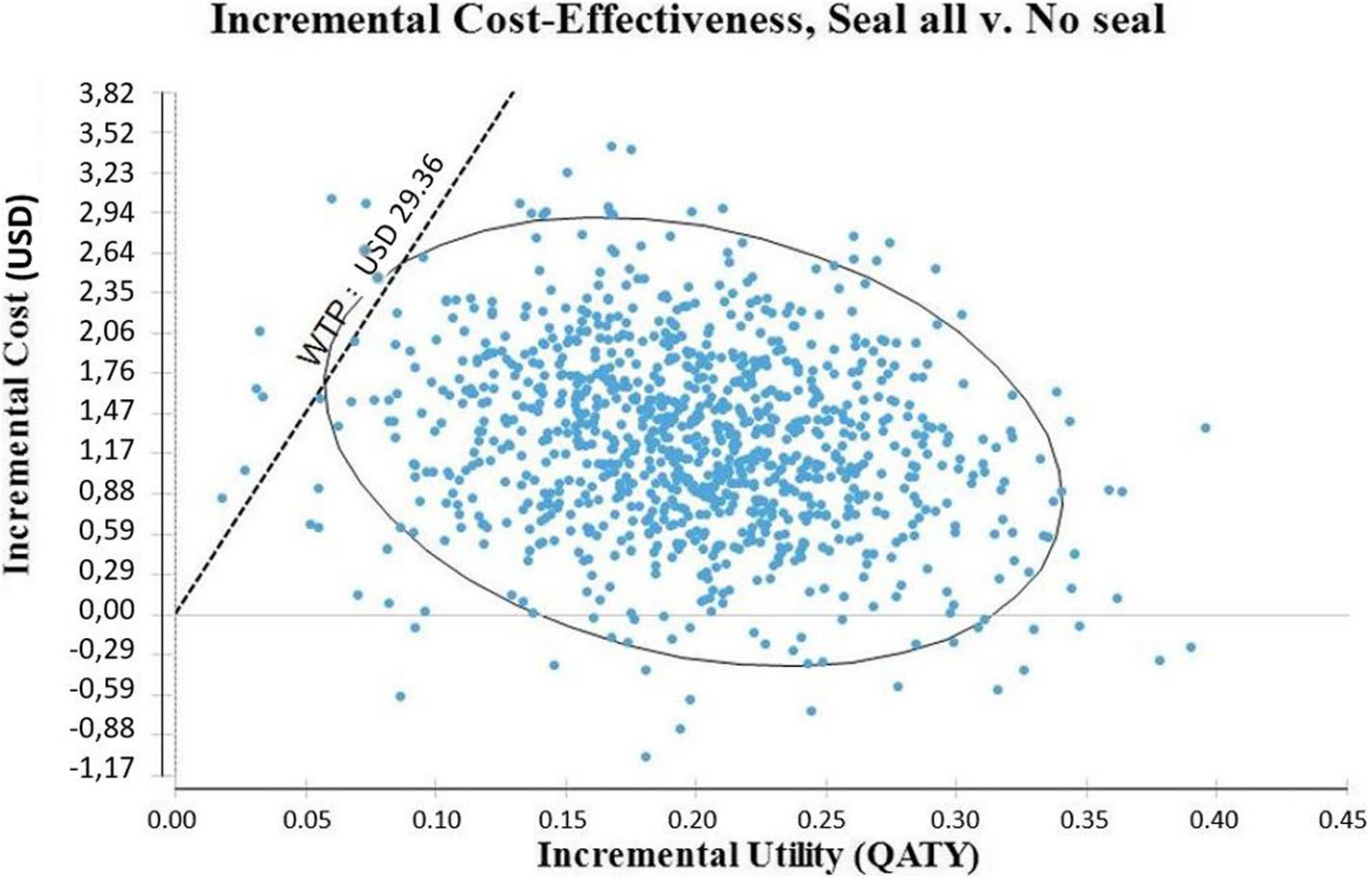
Probabilistic sensitivity analysis

Figure 5 shows the probabilistic sensitivity analysis (Monte Carlo simulation) with 1000 iterations, simulating 1000 patients; it considered the simultaneous influence of all the variables. In 0.9% of cases the “seal all” strategy did not produce a utility, considering a threshold of USD 29.36 (CLP 20,000). However, this strategy was only dominant (i.e. more effective and less expensive) in 3.3% of cases. In the 95.8% of the cases, it presented different levels of cost-utility, mainly concentrated around the mean utility of 0.2 QATY.

## Discussion

The present analysis model showed that the universal application of resin sealants in the context of school-based sealant programs would be a cost-effective measure in populations where the prevalence of caries in first permanent molars is high (USD 6.48 per QATY, CLP 4412 per QATY). This agrees with the findings reported by Bertrand et al. [10] using a model in which the mouth rather than the molar, was the unit of analysis. That study also considered indirect costs, and reached similar conclusions with respect to the utility of sealants in schools. Indeed, the authors note that in comparison with other sealing strategies, dental clinics associated with schools are the option that provides the highest utility.

The sensitivity analysis highlights the fact that the variable with the greatest influence on the ICUR was the increment in caries in first permanent molars. The “seal all” strategy became dominant when the increase in the prevalence of caries in first permanent molars was greater than the base case, by at least 3%. This is in accordance with what was reported by Griffin et al. [39] in their economic evaluation about school-based sealant programs in low-income schoolchildren in the United States. They assumed a similar increment of caries to our scenario and they concluded that this variable was the most influential in the cost-effectiveness ratio. They also concluded that school-based sealant programs saved society money and remained cost-effective across a wide range of reasonable values. Other studies also confirm that sealants present better utility than fillings, when applied to children with a high prevalence of caries [12, 31].

Another variable that creates uncertainty is the reseal rate. When a sealed molar is scheduled for follow up, and the sealant is missing, there are additional costs to consider for the application of a new sealant. In circumstances where the sealant repetition rate is high, ICUR increases considerably. This was also reported by Chi and collaborators, when the retention rate was 10% higher than the base case, the cost was less than half, per averted filling [40].

One strength of this study is the use of multivariate probabilistic analysis, which considers the effect of all variables simultaneously. This generates a model that better resembles the real setting; including the willingness to pay as a reference; and uses a Monte Carlo simulation, which shows a probability that the intervention cost will result in a return of utility. The probability that the “seal all” strategy will not present a utility is only 0.9%. In 3.3% of cases the “seal all” strategy was dominant, that is a higher utility and lower cost.

This model included the opportunity for children to repeat the treatment at their two-year check-ups in cases where the sealant was missing partially or totally lost, increasing the benefit of sealing. However, this implies an incremental cost in this strategy; as explained above, because an excessive re-seal rate may triple the cost per QATY.

The present model also included aspects which need to be considered, as they are part of health states and have often been omitted from prior studies, such as two yearly dental check-ups, oral health education and fluoride treatment, tooth loss for other causes, costs and probabilities of new fillings when treatments fail.

When considering the results of this study, it should be recognized that it is a theoretical model, in which both costs and effectiveness were estimated, some from the literature and others from general market conditions. However, to minimize this limitation, we reviewed the literature to include the best evidence available about the effectiveness of resin-based sealants. Furthermore, the scope of this study was limited to the application of pit and fissure sealants, without considering any added benefits for the children enrolled in school-based sealant programs.

Since our setting considered the scenario of a Chilean school-based sealant program, where all first permanent molars were sealed, a risk-based sealing analysis was not included. Evidence shows that cost-effectiveness of risk-based strategies depend on their prevalence. Studies have shown that the “seal all” strategy becomes more favorable when compared with sealing only patients with high risk of caries [9, 27]. Other authors have concluded that sealants present a greater utility when applied to high-risk patients only [10, 11, 41]. Nonetheless, costs increased when the application was generalized and included unnecessarily sealing molars in children with low risk of caries. On the other hand, there are probably a number of high-risk children who do not receive treatment, due to a lack of dental coverage, which increases their prevalence of caries [12].

Another aspect to consider is that some probabilities were not obtained from the literature, but from a panel of experts. These included the percentage of sealants and fillings that failed in each cycle, and the number of teeth extractions due to caries or other causes. Although, the probabilities of an economic evaluation should be based on clinical data, when this information is not available, the opinion of experts can guarantee that the model reflects real-life practice [42]. The average re-sealing estimated by experts, was similar to that reported in the literature [6]. However, the re-filling parameter reported by experts was lower than that reported in the literature [28, 29], but the latter was considered in the sensitivity analysis.

Most of these events do not influence the ICUR, with the exception of the re-sealing and re-filling rate. Consequently, in the sensitivity analysis we used a broad range that included resealing and re-filling rates used in other economic evaluations and thus assessing the real impact on the model. According to our knowledge, this is the first economic evaluation about school-based sealant programs that included the probabilities of teeth extractions due to caries or other causes, these have not been previously estimated in other economic evaluations.

On the other hand, the root canal treatment was not included in our study, due to the perspective used (i.e., public payer). The few studies that have included root canal treatment as a consequence show this doesn’t change the direction of the cost-effectiveness analysis, since this cost is higher in the group without sealants [31].

Future studies should include long-term costs and expenses, the benefits shown by quality of life determinants, analysis of the risk sub-groups, and the patients’ point of view. This would allow a greater understanding of the social utility of this treatment, as proposed by Kitchens [43]. Additionally, in Chile public and private costs are very different, so it may also be interesting to consider these differences in future analysis.

## Conclusions

This study provides evidence of the cost-utility of school-based sealant programs in children with high prevalence of caries, in the conditions prevailing in Chile. Considering that caries has an important social gradient, this evidence supports public policies of targeting state resources to finance comprehensive oral health care. These policies should include the application of sealants in children from low-income families where caries risk is high. In this context, the investment in oral health would be even more cost-effective and would be evident in a greater number of quality-adjusted tooth years.

## Supplementary information

**Additional file 1.** Detail of the costs considered in the model.

## Data Availability

The datasets used and/or analyzed during the current study are available from the corresponding author upon reasonable request.

## Abbreviations

FPM: First permanent molars
ICUR: Incremental cost-utility ratio
QATY: Quality-adjusted tooth years
