# Supplementary material for: The cost-utility of school-based first permanent molar sealants programs: a Markov model
Source: BMC Oral Health. 2019 Dec 30;19:293. doi: 10.1186/s12903-019-0990-3 (PMC6937712; doi:10.1186/s12903-019-0990-3)
Supplement: Supplementary file 1 — Additional file 1. Detail of the costs considered in the model. [file 12903_2019_990_MOESM1_ESM.pdf]

ADDITIONAL FILE 1

1.- Human Resource Costing

Human resource (Average value)	Monthly salary	Hours per month	Minutes per month	Value of the minute	Adjustment of the IPC May 2019
Dentist	USD 2,257	176	10,560	USD 0.21	USD 0.24
Dental assistant	USD 636	176	10,560	USD 0.06	USD 0.07

Human resource (Lower limit)	Monthly salary	Hours per month	Minutes per month	Value of the minute	Adjustment of the IPC May 2019
Dentist	USD 1,913	176	10,560	USD 0.18	USD 0.2
Dental assistant	USD 532	176	10,560	USD 0.05	USD 0.06

Human resource (Upper limit)	Monthly salary	Hours per month	Minutes per month	Value of the minute	Adjustment of the IPC May 2019
Dentist	USD 2,601	176	10,560	USD 0.25	USD 0.28
Dental assistant	USD 806	176	10,560	USD 0.08	USD 0.09

2. Costing of supplies

Dental Sealants

Dental supplies	Dental supplier 1	Dental supplier 2	Dental supplier 3	Mean
Cotton	USD 14.81	USD 20.53	USD 19.09	USD 18.14
Saliva Ejector	USD 2.53	USD 2.92	USD 2.50	USD 2.65
Gloves	USD 5.13	USD 4.98	USD 4.55	USD 4.89
Masks	USD 3.67	USD 2.92	USD 2.35	USD 2.98
Orthophosphoric acid	USD 2.16	USD 2.19	USD 2.06	USD 2.14
Dental Sealant	USD 14.69	USD 13.87	USD 17.33	USD 15.30

Costing

Dental supplies	Presentation	Consumption by intervention		Average Value	Lower value	Higher Value
Cotton	Package 2000 units	6/1	6	USD 0.05	USD 0.04	USD 0.06
Saliva Ejector	Package 100 units	1/1	1	USD 0.03	USD 0.02	USD 0.03
Gloves	Box 100 units	6/3	0,50	USD 0.05	USD 0.05	USD 0.05
Masks	Box 100 units	6/4	0,125	USD 0	USD 0	USD 0
Orthophosphoric acid	Syringe x 4 ml	6/5	0,016667	USD 0.04	USD 0.03	USD 0.04
Dental Sealant	Syringe	6/6	0,016667	USD 0.25	USD 0.23	USD 0.29
Total				USD 0.42	USD 0.38	USD 0.47
Adjustment of the IPC May 2019				USD **0.47**	USD **0.43**	USD **0.53**

Composite resin

Dental supplies	Dental supplier 1	Dental supplier 2	Dental supplier 3	Mean
Disposable needles	USD 5.91	USD 6.15	USD 6.46	USD 6.17
Cotton	USD 14.81	USD 20.53	USD 1.91	USD 18.14
Anesthesia	USD 12.68	USD 14.54	USD 15.56	USD 14.26
Composite sandpaper	USD 2.05	USD 1.22	USD 1.06	USD 1.44
Saliva ejector	USD 2.53	USD 2.92	USD 2.50	USD 2.65
Dental wedges	USD 8.14	USD 9.37	USD 13.80	USD 10.44
Diamond burs	USD 1.01	USD 0.87	USD 1.01	USD 0.96
Composite polishing burs	USD 14.68	USD 14.68	USD 14.68	USD 14.68
Gloves	USD 5.13	USD 4.98	USD 4.55	USD 4.89
Calcium hydroxide	USD 11.41	USD 11.41	USD 3.23	USD 8.68
Glass ionomer Liner	USD 18.65	USD 17.47	USD 17.62	USD 17.92
Masks	USD 3.67	USD 2.92	USD 2.35	USD 2.98
Polishing disc	USD 0.88	USD 0.88	USD 0.88	USD 0.88
Composite resin	USD 93.03	USD 105.65	USD 93.38	USD 97.35
Adhesive	USD 0.00	USD 27.88	USD 19.09	USD 23.48
Celluloid strips	USD 4.71	USD 1.31	USD 1.91	USD 2.64
Orthophosphoric acid	USD 2.16	USD 2.19	USD 2.06	USD 2.14
Articulating paper	USD 6.97	USD 6.59	USD 5.29	USD 6.28

Costing

Dental supplies	Presentation	Consumption by intervention		Average Value	Lower value	Higher Value
Disposable needles	Boxs de 100 units	1/2	0,5	USD 0.03	USD 0.03	USD 0.03
Cotton	Package 2000 units	6/1	6	USD 0.05	USD 0.04	USD 0.06
Anesthesia	Box por 50 units	1/2	0,5	USD 0.14	USD 0.13	USD 0.16
Composite sandpaper	Boxs de 25 units	1/2	0,5	USD 0.03	USD 0.02	USD 0.04
Saliva ejector	Package 100 units	1/1	1	USD 0.03	USD 0.02	USD 0.03
Dental wedges	Package 200 units	1/2	0,5	USD 0.03	USD 0.02	USD 0.03
Diamond burs	Unit	1/20	0,05	USD 0.05	USD 0.04	USD 0.05
Composite polishing burs	Unit	1/80	0,0125	USD 0.18	USD 0.18	USD 0.18
Gloves	Box of 50 pairs	1/4	0,25	USD 0.02	USD 0.02	USD 0.03
Calcium hydroxide	Kit	1/200	0,005	USD 0.04	USD 0.02	USD 0.06
Glass ionomer liner	Kit	1/200	0,005	USD 0.09	USD 0.09	USD 0.09
Masks	Box of 100 units	1/12	0,083333	USD 0.002	USD 0.002	USD 0.003
Polishing disc	Unit	1/20	0,05	USD 0.04	USD 0.04	USD 0.04
Composite resin	4 Syringes	1/200	0,005	USD 0.49	USD 0.47	USD 0.53
Adhesive	Bottle	1/200	0,005	USD 0.12	USD 0.1	USD 0.14
Celluloid strips	Box 100 units	1/2	0,5	USD 0.01	USD 0.01	USD 0.02
Orthophosphoric acid	Syringe x 4 ml	1/50	0,0125	USD 0.03	USD 0.03	USD 0.03
Articulating paper	Box of 12 block	1/200	0,005	USD 0.03	USD 0.03	USD 0.03
Total				USD 1.42	USD 1.29	USD 1.56
Adjustment of the IPC May 2019				USD **1.59**	USD **1.44**	USD **1.75**

Dental Extraction

Dental supplies	Dental supplier 1	Dental supplier 2	Dental supplier 3	Mean
Disposable needles	USD 5.91	USD 6.15	USD 6.46	USD 6.17
Cotton	USD 14.81	USD 20.53	USD 19.09	USD 18.14
Anesthesia	USD 12.68	USD 14.54	USD 15.56	USD 14.26
Masks	USD 3.67	USD 2.92	USD 2.35	USD 2.98
Gloves	USD 5.13	USD 4.98	USD 4.55	USD 4.89
Saliva Ejector	USD 2.53	USD 2.92	USD 2.50	USD 2.65

Costing

Dental supplies	Presentation	Consumption by intervention		Average Value	Lower value	Higher Value
Disposable needles	Boxs de 100 units	1/1	1	USD 0.06	USD 0.06	USD 0.06
Cotton	Package 2000 units	6/1	6	USD 0.05	USD 0.04	USD 0.06
Anesthesia	Box 50 units	1/1	1	USD 0.29	USD 0.25	USD 0.31
Masks	Box 100 units	1/12	0,083333	USD 0	USD 0	USD 0
Gloves	Box 100 units	1/1	1	USD 0.1	USD 0.09	USD 0.1
Salva ejector	Package 100 units	1/1	1	USD 0.03	USD 0.02	USD 0.03
Total				USD 0.53	USD 0.48	USD 0.57
Adjustment of the IPC May 2019				USD **0.59**	USD **0.53**	USD **0.64**

Oral Exam

Dental supplies	Dental supplier 1	Dental supplier 2	Dental supplier 3	Mean
Cotton	USD 17.74	USD 20.53	USD 19.09	USD 19.12
Masks	USD 3.67	USD 2.92	USD 2.64	USD 3.08
Gloves	USD 5.13	USD 4.98	USD 4.70	USD 4.94
Saliva ejector	USD 2.53	USD 2.92	USD 2.64	USD 2.70

Costing

Dental supplies	Presentation	Consumption per intervention		Mean Value	Lower Value	Higher Value
Cotton	Package 2000 units	6/1	8	USD 0.06	USD 0.05	USD 0.06
Masks	Box 100 units	1/2	0.333	USD 0.01	USD 0.01	USD 0.01
Gloves	Box 100 units	1/1	2	USD 0.1	USD 0.09	USD 0.1
Dental ejector	Package 100 units	1/1	1	USD 0.03	USD 0.02	USD 0.03
**Total**				USD 0.19	USD 0.18	USD 0.2
Adjustment of the IPC May 2019				USD **0.22**	USD **0.2**	USD **0.23**
